# Mechanistic Studies on the Stereoselectivity of FFAR1 Modulators

**DOI:** 10.1021/acs.jcim.2c00417

**Published:** 2022-07-25

**Authors:** Dan Teng, Yang Zhou, Yun Tang, Guixia Liu, Yaoquan Tu

**Affiliations:** † Shanghai Frontiers Science Center of Optogenetic Techniques for Cell Metabolism, Shanghai Key Laboratory of New Drug Design, School of Pharmacy, East China University of Science and Technology, Shanghai 200237, China; ‡ Department of Theoretical Chemistry and Biology, School of Engineering Sciences in Chemistry, Biotechnology and Health (CBH), KTH Royal Institute of Technology, Stockholm SE-106 91, Sweden; § School of Pharmacy, Jinan University, 601 Huangpu Avenue West, Guangzhou 510632, China

## Abstract

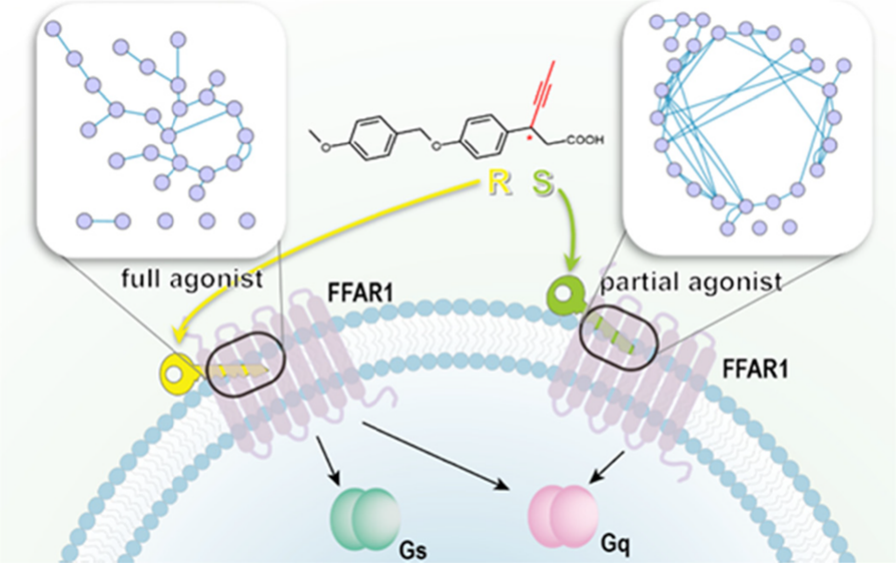

Free fatty acid receptor 1 (FFAR1) is a potential therapeutic target for the treatment of type 2 diabetes (T2D). It has been validated that agonists targeting FFAR1 can achieve the initial therapeutic endpoints of T2D, and the epimer agonists (*R*,*S*) AM-8596 can activate FFAR1 differently, with one acting as a partial agonist and the other as a full agonist. Up to now, the origin of the stereoselectivity of FFAR1 agonists remains elusive. In this work, we used molecular simulation methods to elucidate the mechanism of the stereoselectivity of the FFAR1 agonists (*R*)-AM-8596 and (*S*)-AM-8596. We found that the full agonist (*R*)-AM-8596 disrupts the residue interaction network around the receptor binding pocket and promotes the opening of the binding site for the G-protein, thereby resulting in the full activation of FFAR1. In contrast, the partial agonist (*S*)-AM-8596 forms stable electrostatic interactions with FFAR1, which stabilizes the residue network and hinders the conformational transition of the receptor. Our work thus clarifies the selectivity and underlying molecular activation mechanism of FFAR1 agonists.

## 1 Introduction

Free fatty acid receptor 1 (FFAR1), the first deorphanized FFAR, is a G-protein-coupled receptor (GPCR) belonging to the rhodopsin family.^1^ FFAR1 is primarily expressed in insulin-secreting pancreatic β-cells and gut enteroendocrine cells and can be activated by medium or long chain fatty acids.^2^ FFAR1 plays a critical role in stimulating the release of incretins on enteroendocrine cells and amplifying the release of insulin on pancreatic β-cells.^3,4^ Because FFAR1 exerts multiple beneficial effects on metabolic syndrome and has a low risk of hypoglycemia, it has attracted considerable attention as an emerging therapeutic target for type 2 diabetes (T2D).^5^ Preclinical and clinical studies suggest that FFAR1 agonists can achieve the initial therapeutic endpoint of T2D, and many academic institutes and pharmaceutical companies are racing to develop FFAR1 agonists. FFAR1 is accepted as one of the most important targets for the treatment of T2D, albeit with a need for further characterization of its binding mode, intracellular signaling, and toxicity.^6^

It has been known that signal transduction generated by stimulation of FFAR1 is tissue-specific. Co-activation of the Gq and Gs proteins is the major pathway to stimulate incretins (GLP-1 and GIP) release in enteroendocrine cells,^7^ while the effect of glucose-stimulated insulin secretion in pancreatic β-cells is mainly mediated by the Gq protein.^8^ Full agonists of FFAR1 engage in both the enteroendocrine signaling axis and the pancreatic β-cell signaling axis. Activation of both pathways ultimately amplifies glucose-stimulated insulin secretion in pancreatic β-cells.^6^ Additionally, GLP-1 has multiple pharmacological and physiological effects, such as inhibition of glucagon secretion and pancreatic β-cell apoptosis, which could further benefit patients with T2D,^9^ while partial agonists such as AMG-837 engage in only the pancreatic β-cell signaling axis. Interestingly, (*R*,*S*)-AM-8596 are a pair of epimers discovered by the Amgen team, and the evaluation results indicate that (*R*)-AM-8596 is a full agonist and (*S*)-AM-8596 is a partial agonist.^10^ This raises a key question of how agonists with similar structures induce distinct signal transduction. The answer to this question can help us design agonists in a targeted manner and give an insight into the allosteric activation mechanism of FFAR1. However, no relevant clues can be found from the current experimental data and available crystal structures.

**Figure 1:**
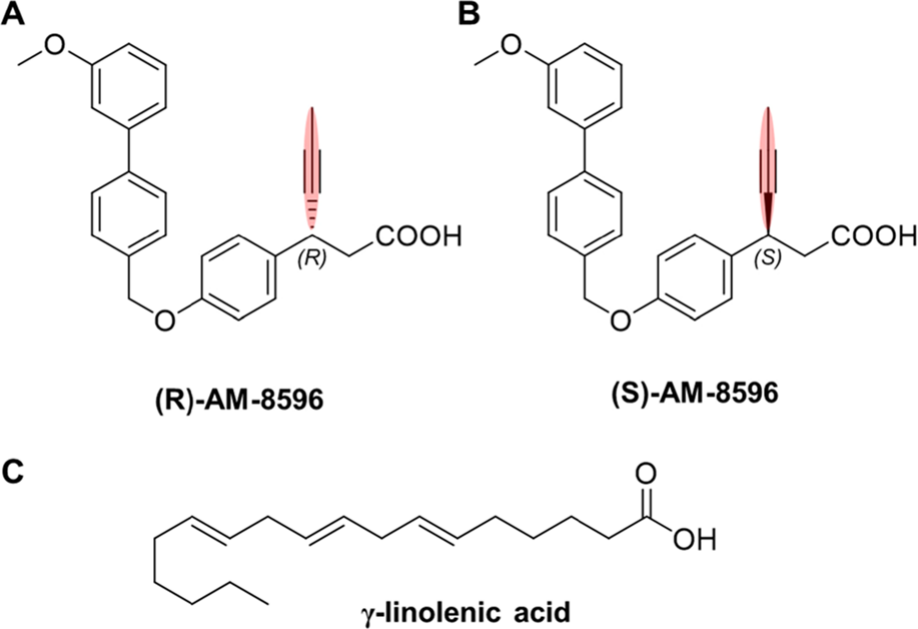
Ligands used in this work. (A) Full agonist (*R*)-AM-8596 and (B) partial agonist (*S*)-AM-8596. The propynyl moiety on the chiral carbon is highlighted in red. (C) Endogenous ligand γ-linolenic acid.

It has been found from the available crystal structures that FFAR1 has two well-defined binding sites. One is the site at which TAK-875 binds^11^ and is located in between transmembrane (TM) helices 3–5 and extracellular loop 2 (ECL2) of FFAR1. The other is the lipid-facing binding site formed by TM4–5 and intracellular loop 2 (ICL2). This site is also considered as the binding site for the endogenous ligand γ-linolenic acid.^12^ To address the structural basis of the stereoselectivity of FFAR1, we first built the complexes of FFAR1 binding to (*R*)-AM-8596, (*S*)-AM-8596, and the endogenous ligand γ-linolenic acid, respectively (Figure 1). Then, long-time atomistic molecular dynamics (MD) simulations were performed for each system, and the binding modes of the two enantiomers in the equilibrium state were identified. We calculated the binding free energies for the enantiomers, which explain well the experimental results. By analyzing the simulation results, we found that the two different agonists exert their effects by affecting the residue interaction network inside the receptor, which results in distinct conformational changes in the overall structure of the receptor. Our work thus clarifies the mechanism of the stereoselectivity of the FFAR1 agonists.

## 2 Materials and Methods

### 2.1 Protein Preparation

The crystal structure of FFAR1 was obtained from the PDB database (PDB ID: 4PHU^11^). For modeling the wild-type receptor, the T4 lysozyme (on intracellular loop 3) and four thermostabilizing point mutations used to facilitate crystal growth and increase structural stability were removed. Then, the two missing residues S212^ICL3^ and G213^ICL3^ on ICL3 were built with Prime,^13^ and the top-ranked refined loop was chosen for protein construction. Four point mutations (L42A^2.40^, F88A^3.34^, G103A^3.49^, and Y202F^5.38^) were restored according to the human protein sequence obtained from UniProt (UniProtKB-O14842). The repaired 3D structure was then imported into Protein Preparation Wizard (Proprep)^14^ to check the protonation states of amino acids His, and no His has been found in the protonated state. In addition, hydrogen atoms were added to the FFAR1 model at the physiological pH value with PROPKA.^15^

### 2.2 Molecular Docking

The docking procedure was carried out with Glide^16,17^ (Schrödinger 2021 suite). It has been shown that the lead compound of (*R*,*S*)-AM-8596 binds at the same site as the co-crystallized ligand TAK-875.^18^ This binding site is denoted as site 1 in this work. The endogenous ligand γ-linolenic acid binds at the lipid-facing binding site formed by TM4–5 and ICL2.^12^ This binding site is denoted as site 2 in this work. Each ligand was sketched in Maestro^19^ and initially placed at the binding site with the pose similar to that of the co-crystallized ligand in the corresponding crystal structure. Before the flexible ligand docking was executed, the center of the box with a size of 18.0 × 18.0 × 18.0 Å^3^ was placed on the center of mass of the ligand. The complex conformation with the best docking score was selected as the initial structure for MD simulations.

### 2.3 MD Preparation

The POPC bilayer was generated with VMD (version 1.9.1),^20^ and the receptor was pre-aligned using the reference structure of FFAR1 obtained from the Orientations of Proteins in Membranes database.^21^ 104 POPC lipids with 10,260 TIP3P water molecules in a cubic box of 75.0 × 75.0 × 100.0 Å^3^ were used to build the protein–membrane system. 59 Cl^–^ and 51 Na^+^ were added to generate the neutral systems with the NaCl concentration of 0.15 M to reproduce the physiological state as much as possible. The CHARMM 36 force field^22^ was used to model the protein, lipids, water molecules, and ions. The parameters for the ligands were determined with the CHARMM CGenFF small molecule force field.^23^

### 2.4 Molecular Dynamics Simulations

All simulations were performed using Gromacs 2016. Each system was first subjected to a 10,000-step energy minimization using the steepest descent algorithm with a force threshold of 1000.0 kJ/mol/nm. Then, the system was gradually heated from 0 to 300 K followed by a 1000 ps MD simulation with the *NVT* ensemble. The system was further simulated for 50 ns using the *NPT* ensemble. During the simulations, both the ligand and the protein backbone were restrained by a harmonic potential with a force constant of 1000 kcal mol^–1^ Å^–2^. Finally, a 1000 ns unrestricted simulation was performed for each system under the *NPT* ensemble (Table 1). The cut-offs for the van der Waals and electrostatic interactions were set to 12 Å. The long-range electrostatic interaction was recovered by the particle mesh Ewald summation method.

**Table 1:** Systems for MD Simulations

system	total simulation time	agonist
FFAR1-(*R*)-AM-8596	3 μs (1 μs per simulation)	(*R*)-AM-8596
FFAR1-(*S*)-AM-8596	3 μs (1 μs per simulation)	(*S*)-AM-8596
FFAR1-γ-linolenic-acid	3 μs (1 μs per simulation)	γ-linolenic acid
FFAR1-apo	3 μs (1 μs per simulation)	

### 2.5 Free Energy Calculation

The multistate Bennett acceptance ration estimator^24^ has been chosen to carry out free energy calculations because of its efficiency and convenience. To obtain a reliable free energy difference related to ligand binding, a thermodynamic cycle was devised. The thermodynamic cycle depicted in Figure S1 contains two sets of calculations, namely, the ligand decoupled from the complex and the ligand decoupled from solution. The coupling parameter λ (also referred to as *windows*) is used to define the thermodynamic states of the system along the alchemical pathway, scaled charges, parameters of Lennard-Jones interactions, and force constants of restraints. The settings of free energy calculations were based on the published work of Aldeghi et al.^25^

For a protein–ligand complex, the use of restraints is important because it prevents the ligand from leaving the binding site when it is not interacting with the environment. This also ensures that conformational sampling during the simulations corresponds to a well-defined bound state and contributes to a good phase space overlap between two neighboring windows and faster convergence.^26,27^ The calculation is performed in three parts as described below.

First, to keep the orientation and position of the decoupled ligand similar to the reference structure, we chose three atoms from the rigid part of the receptor (backbone) and three atoms from an agonist in the starting structure (Figure S2). The starting structures for the calculations were extracted from the equilibrium phase of the simulations. Six constraints [one for distance (*d*), two for angles (θ_A_ and θ_B_), and three for dihedral angles (φ_A_, φ_B_, and φ_C_)] were imposed on the chosen atoms to restrain the six degrees of freedom between the protein and the ligand. The selected atoms, reference distances, and angles are presented in Table S1. Here, 11 windows were used, which correspond to λ_restr_ = 0.00, 0.01, 0.025, 0.05, 0.075, 0.10, 0.20, 0.35, 0.50, 0.75, and 1.00. The free energy of restraining the decoupled ligand to a certain pose can be calculated analytically using the approach proposed by Boresch et al.^26^ The following potential form (eq 1) was used for structural restraints,
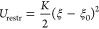
1where ξ_0_ is the reference value, ξ is a specific parameter to be restrained, and *K* is the force constant for the harmonic restraint, with *K* = 10.0 kcal mol^–1^ Å^2^ for the distance or *K* = 10.0 kcal mol^–1^ rad^2^ for an angle or dihedral.

In the second part of the calculation, the electrostatic interactions of the ligand were decoupled from the receptor. Five evenly spaced λ values, with λ_coul_ = 0.00, 0.25, 0.50, 0.75, and 1.00, were used to decouple the ligand charges.

In the last part, the soft-core potential was used to decouple the Lennard-Jones interactions between the ligand and the receptor.^28^ Here, 16 λ_vdw_ values were used (λ_vdw_ = 0.00, 0.05, 0.10, 0.20, 0.30, 0.40, 0.50, 0.60, 0.65, 0.70, 0.75, 0.80, 0.85, 0.90, 0.95, and 1.00), with the intramolecular interactions not decoupled.

For the ligand decoupled from the solution, we only turned off the electrostatic and Lennard-Jones interactions and used the same windows as stated above. For each state defined by a λ value, energy minimization and equilibration were carried out. This means that for each of the 50 λ windows, we performed 1000 steps of energy minimization with the steep descent method, a 100 ps MD simulation using the *NVT* ensemble (with *T* = 300 K), a 200 ps MD simulation using the *NPT* ensemble (with *P* = 1 atm and *T* = 300 K) for equilibration, and a production simulation for 15 ns using the same *NPT* ensemble.

### 2.6 Analysis of the Simulation Results

The alchemical analysis tool,^29^ a Python program that implements an automated analysis of free energy calculations performed with several simulation engines (such as Gromacs and Amber), was used to analyze the data collected from all simulations.

The characterization of the interactions between the protein and the ligand was performed using the PLIP tool.^30^ Here, PLIP was used to analyze the structures extracted from the simulation trajectories, and the results were further studied using an in-house program.

The residue interaction network of the receptor was calculated with RING, a tool for generating contact maps from protein structures,^31^ and visualized with Cytoscape.^32^ The average structures, which were obtained with the cluster analysis tool in Gromacs based on the last 500 ns of the MD trajectory, were introduced into the RING for analysis. The RING algorithm generates a network for the interactions between the protein residues in two steps. First, a list of residue–residue pairs were determined based on the distance measurement. Then, contact characteristics were identified based on the type of interactions. In this study, we first employed the “Closest” strategy to measure the shortest distance between a pair of residues. In the second step, the “Multiple” type parameter was used to identify the interaction types for each pair. The shortest path distance between two residues in the network is also referred to as the minimum number of nodes that needs to be traversed from one residue to another. Here, NAPS was employed for residue shortest path analysis.^33^

To characterize the accessibility of the G-protein to the intracellular cavity of FFAR1, we first determined the atlas surface topography of the G-protein cavity in the complex of the β_2_ adrenergic receptor (β_2_AR) and G-protein (PDB:3SN6) using CASTp.^34^ Then, by aligning the FFAR1 to the structure of β_2_AR, 45 equivalent residues were identified (Figure S3). Finally, the solvent accessible surface area (SASA) of the G-protein cavity was calculated using the SASA tool of Gromacs. In addition, the distance and secondary structure information of the receptor was obtained using the distance and do_dssp tools in Gromacs, respectively. The stability of each ligand during the simulation was analyzed based on its root mean square deviation (RMSD) value. The *B*-factors of the receptor were calculated using the rmsf command in Gromacs.

## 3 Results and Discussion

To explain the structural basis of FFAR1 stereoselectivity, we first built the complexes of FFAR1 binding to (*R*)-AM-8596 and (*S*)-AM-8596, respectively, based on the crystal structure of FFAR1 (PDB ID: 4PHU).^11^ (*R*,*S*)-AM-8596 have the binding modes similar to TAK-875.^18^ The docking results indicate that (*R*,*S*)-AM-8596 indeed form strong electrostatic interactions with two key residues R183^5.39^ and R258^7.35^ in the binding pocket. Then, 3 1 μs MD simulations with different initial velocities were performed for each complex system. From the trajectory of the product run, a representative structure was selected for the estimation of the binding free energy of the ligand to the receptor. The results show that the binding free energies of (*R*)-AM-8596 and (*S*)-AM-8596 to FFAR1 are −11.102 and −12.457 kcal mol^–1^, respectively, with the binding of (*S*)-AM-8596 to the receptor being more stable than that of (*R*)-AM-8596 (Table 2). According to the functional experiments, the EC_50_ of the full agonist (*R*)-AM-8596 is 3.8 ± 0.54 μM, and the EC_50_ of the partial agonist (*S*)-AM-8596 is 0.65 ± 0.03 μM, suggesting that (*S*)-AM-8596 seems to have a higher binding affinity.^10^ Our results thus indicate that for this pair of agonists, the activation effect is most likely correlated to the binding affinity of the agonist.

**Table 2:** Binding Free Energy Components of the Two Agonists (kcal mol^–1^)

ligand	–Δ*G*_elec + vdw_^int_complex^	Δ*G*_elec + vdw_^int_water^	Δ*G*_restr_on_	Δ*G*_bind_
(*R*)-AM-8596	–121.602 ± 0.524	103.941 ± 0.254	6.559	–11.102 ± 0.582
(*S*)-AM-8596	–123.153 ± 0.310	103.996 ± 0.213	6.7	–12.457 ± 0.376

### 3.1 Analysis of the Binding Modes

**Figure 2:**
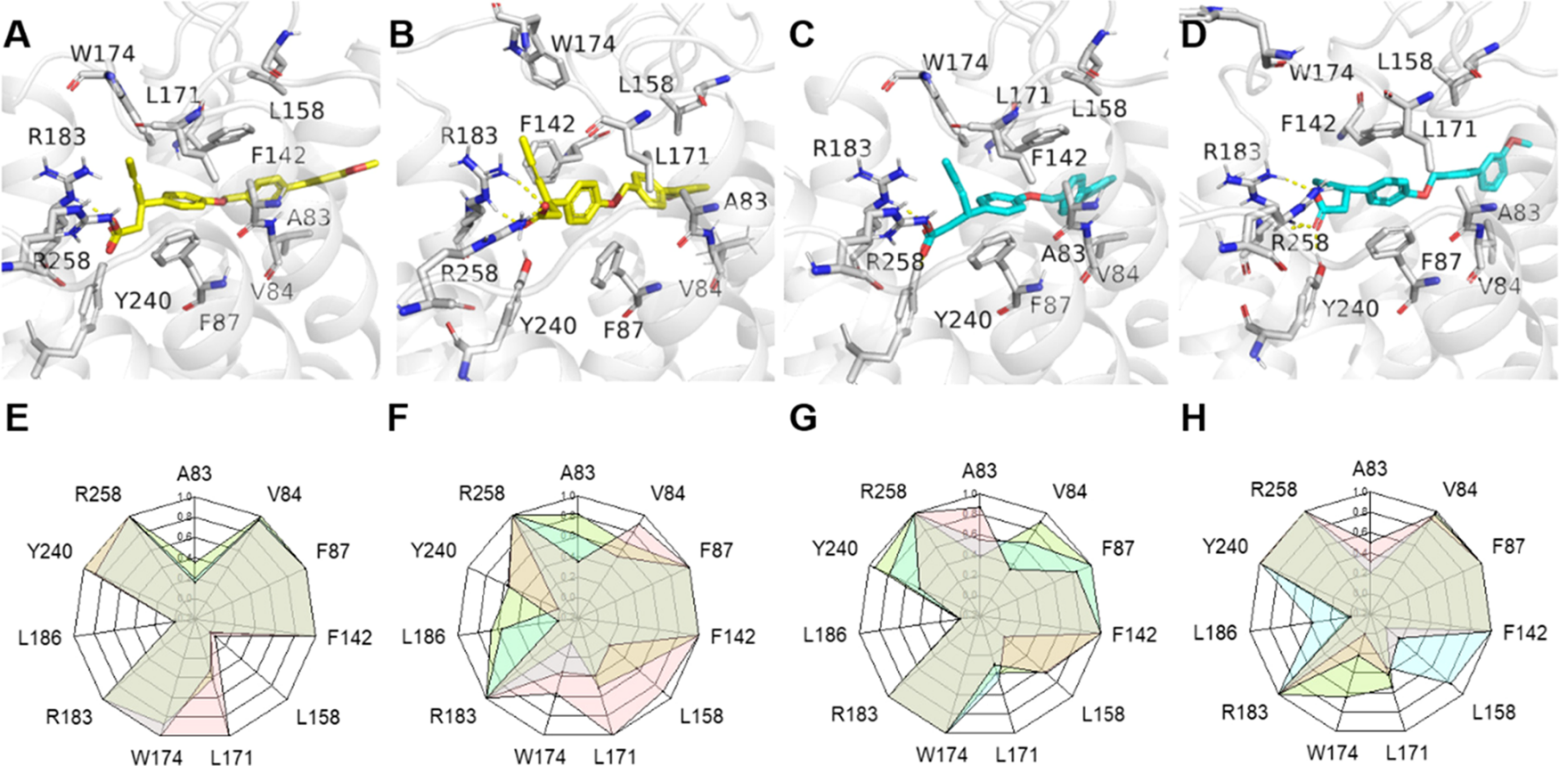
Binding modes of the ligands to FFAR1. (*R*)-AM-8596 and (*S*)-AM-8596 are shown in yellow and cyan, respectively, and the key residues in the receptor binding pocket are highlighted in gray. (A) Initial structure (*t* = 0) and (B) last structure (*t* = 1000 ns) for (*R*)-AM-8596 binding to the key residues of the receptor, obtained from the MD simulation. (C) Initial structure (*t* = 0) and (D) last structure (*t* = 1000 ns) for (*S*)-AM-8596 binding to the key residues of the receptor. The interaction fingerprint between (*R*)-AM-8596 and FFAR1 for the initial 200 ns (E) and the last 200 ns (F) of the three parallel simulations. Interaction fingerprint between (*S*)-AM-8596 and FFAR1 for the initial 200 ns (G) and the last 200 ns (H) of the three parallel simulations.

**Figure 3:**
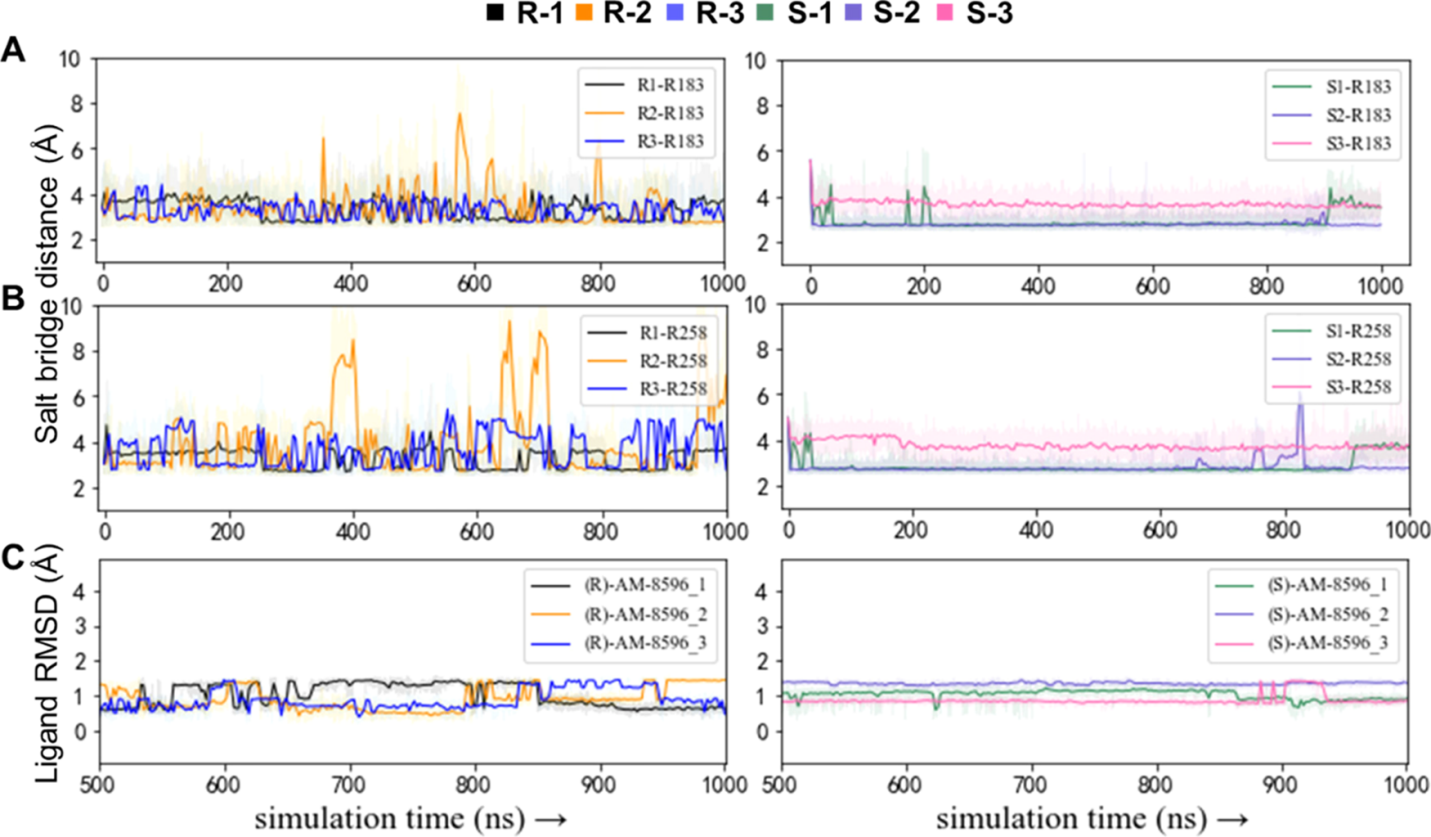
Salt bridge distances between the ligand carboxylic group and the protonated nitrogen on the side chain of R183^5.39^ (A) and R258^7.35^ (B). Black, orange, and blue lines represent the salt bridge distances measured in the three parallel simulations for the (*R*)-AM-8596-bound system, and green, purple, and pink lines represent the salt bridge distances measured in the three parallel simulations for the (*S*)-AM-8596-bound system, respectively. (C) RMSD values of the agonists (*R*)-AM-8596 (left) and (*S*)-AM-8596 (right) in the three parallel simulations.

Since ligand binding is a critical step for a GPCR activation, we first analyzed the binding modes of the (*R*,*S*)-AM-8596 epimers to the FFAR1. By examining the trajectories from the MD simulations, we found that both (*R*)-AM-8596 and (*S*)-AM-8596 form salt bridge interactions with R183^5.39^ and R258^7.35^ (Figure 2A–D). Such salt bridge interactions have also been observed in the crystal structures 4PHU^11^ and 5TZR.^35,36^ The computational and experimental studies on FFAR1 have shown that residues R183^5.39^ and R258^7.35^ are critical for the activity of agonists bound at site 1.^37,38^ Compared with (*R*)-AM-8596, (*S*)-AM-8596 forms stronger salt bridge interactions with R183^5.39^ and R258^7.35^ (Figure 3A,B) (Table S2), which is most likely the main reason why (*S*)-AM-8596 has a higher binding affinity to the FFAR1 than (*R*)-AM-8596. In this work, R183^5.39^ and R258^7.35^ act like anchors and play a critical role in the ligand binding to FFAR1. The interaction fingerprints obtained from the MD simulations (Figure 2E–H) show that the bound (*R*)-AM-8596 forms strong π–π interactions with F87^3.33^ and F142^4.61^ and hydrophobic interactions with A83^3.29^, V84^3.30^, V141^4.60^, L158^ECL2^, L171^ECL2^, and L186^5.44^. Compared with (*R*)-AM-8596, the bound (*S*)-AM-8596 also forms strong π–π interactions with F87^3.33^ and F142,^4.61^ but the hydrophobic interactions formed with the same hydrophobic residues are weaker. In the two epimer complexes, the sandwich-like π–π stacking formed by the benzene ring of the ligand head with F87^3.33^ and F142^4.61^ can stabilize the ligand very well.

Next, we further investigated the differences in the two binding modes. In the FFAR1-(*R*)-AM-8596, the propynyl moiety of (*R*)-AM-8596 faces the ECL2 and forms stronger hydrophobic interactions with L158^ECL2^ and L171^ECL2^ on ECL2 as compared to (*S*)-AM-8596 (Figure 2B). Due to the hydrophobic interactions formed between (*R*)-AM-8596 and the residues on ECL2, the salt bridges formed between (*R*)-AM-8596 and FFAR1 are more easily affected by the fluctuation of ECL2. In the FFAR1-(*S*)-AM-8596 complex, the propynyl moiety of (*S*)-AM-8596 was deflected toward TM4 and TM5, and the propynyl group was trapped in the gap in between TM4 and TM5, which makes the salt bridge interaction between (*S*)-AM-8596 and FFAR1 more stable (Figure 2D). In addition, the ligand RMSD values also indicated that (*S*)-AM-8596 is more stable than (*R*)-AM-8596 at the binding site of FFAR1 (Figure 3C). Therefore, the difference in the orientation of the epimer propynyl moieties could affect the stability of a ligand in the receptor binding pocket.

### 3.2 Analysis of the Residue Interaction Network for the Receptor

**Figure 4:**
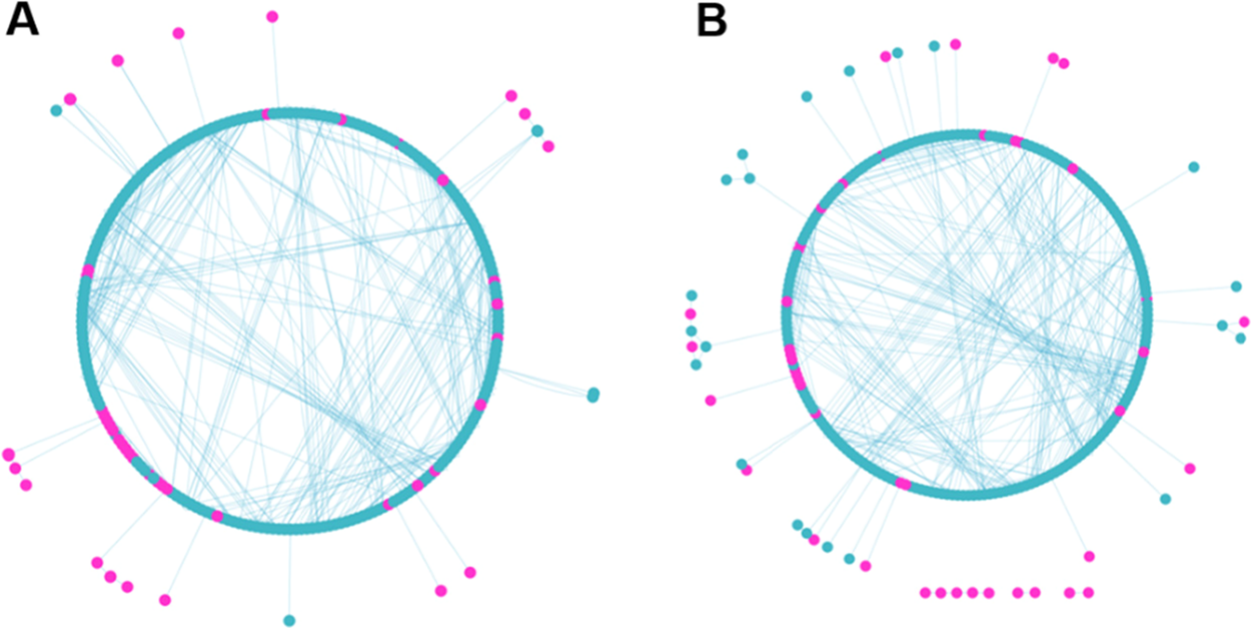
Residue interaction networks. Nodes in a network represent residues, and edges represent interactions between residues. Residues on a helix and loop are shown with blue and pink dots, respectively. (A) Residue interaction network in the (*S*)-AM-8596-bound receptor, with the large circle indicating multiple residue interactions. (B) Residue interaction network in the (*R*)-AM-8596-bound system, with the small circle and more scattered dots indicating weaker residue interactions.

In Figure 4, we illustrate the residue interaction networks of the receptor, in which the large circle indicates multiple residue interactions, and nodes and edges represent residues and interactions formed between residues, respectively. As we can see from Figure 4A, in the partial agonist (*S*)-AM-8596-bound receptor, most of the residues form firm contacts with multiple neighbors, with 256 residues forming 533 interactions. However, in the full agonist (*R*)-AM-8596-bound receptor, the interactions between the residues in the binding pocket were disrupted due to the shift of the helices accompanying the binding of (*R*)-AM-8596 to the receptor and the number of interactions decreases to 499, which are characterized by a smaller number of residues participating in the formation of the main network and more scattered local groups and dots (see Figure 4B).

**Figure 5:**
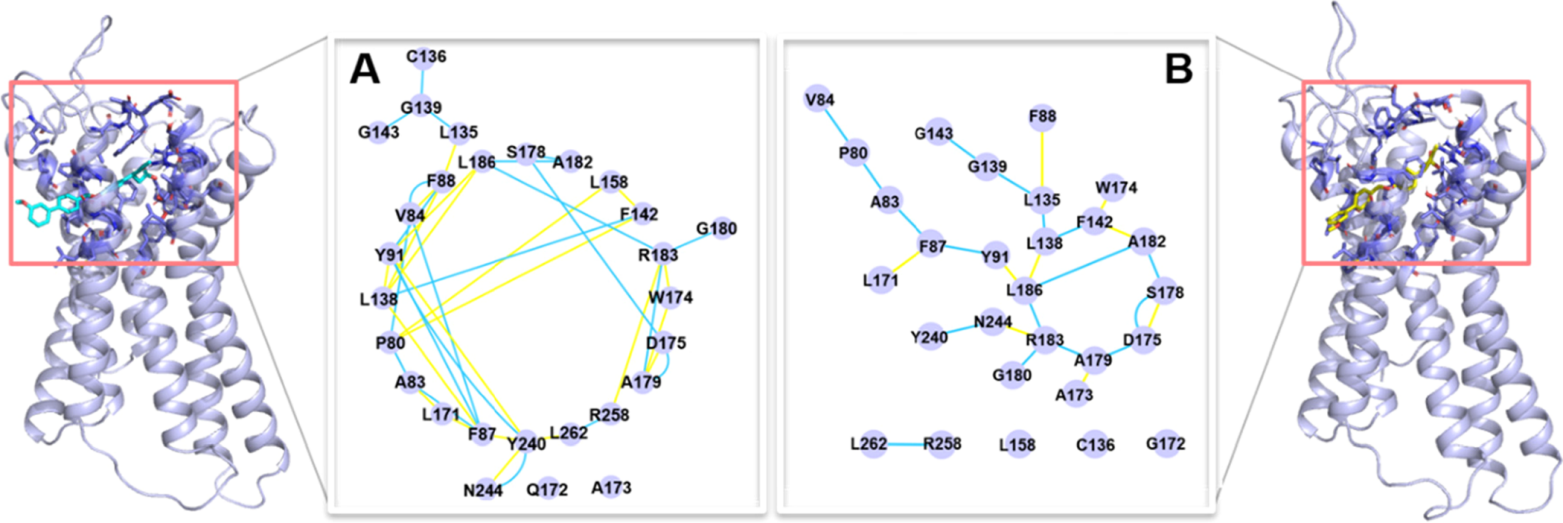
Interaction networks of residues within 5 Å of (A) partial agonist (*S*)-AM-8596 (in cyan) and (B) full agonist (*R*)-AM-8596 (in yellow). The blue and yellow edges represent hydrogen-bond and van der Waals interactions, respectively.

Focusing on the residues within 5 Å of each agonist, we found that the receptor structure due to the binding of the partial agonist is rather different from that due to the binding of the full agonist. In the partial agonist (*S*)-AM-8596-bound network, several key residues of the receptor, such as R183^5.39^, Y240^6.51^, and R258^7.35^, were found to form salt bridges and hydrogen-bond interactions with the ligand and an extensive and very stable hydrogen-bond network with other residues around the ligand (Figure 5A). In the full agonist (*R*)-AM-8596-bound receptor, however, the hydrogen-bond interactions between the residues in the receptor binding pocket were extensively disrupted (Figure 5B). In particular, R258^7.35^, a pivotal residue at the center of the binding site, is almost isolated from the main part of the network. Therefore, we can conclude that the key residues that behave differently in the binding modes of (*R*)-AM-8596- and (*S*)-AM-8596-bound systems may change the overall receptor structure through affecting the stability of the formed hydrogen-bond interaction network. Our study thus suggests that compared with partial agonist (*S*)-AM-8596, full agonist (*R*)-AM-8596 can disrupt the interactions between the residues in the receptor binding pocket to a greater extent, which is key to the structural change of the receptor. The difference in the receptor structure due to the binding of different agonists may be the main reason for the difference in agonist-induced downstream signaling pathways.

### 3.3 Dynamic Structural Changes of the Receptor

**Figure 6:**
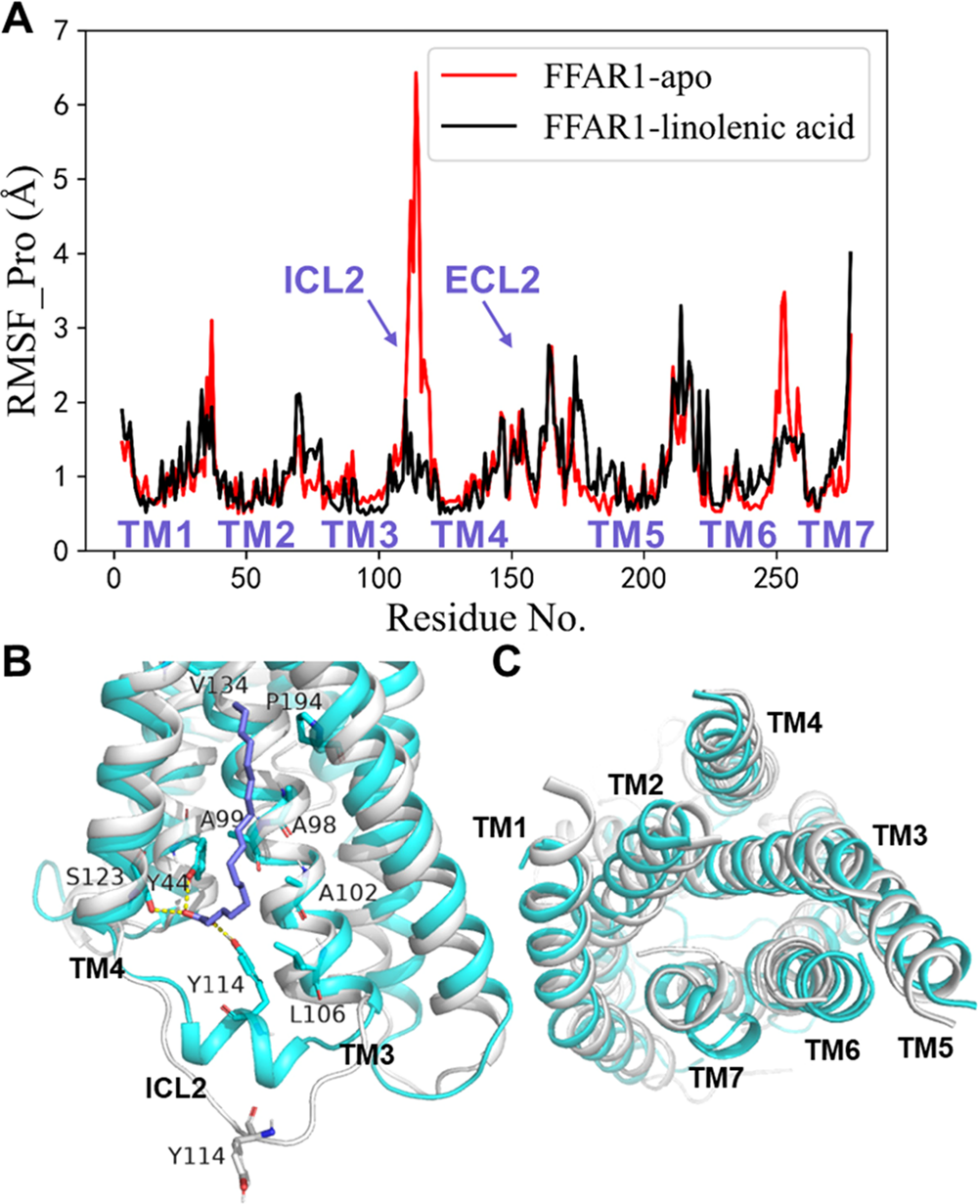
(A) RMSF values of the FFAR1 Cα-atoms obtained from the MD simulations. (B) Superposition of the FFAR1-apo and FFAR1-γ-linolenic-acid complex structures in ribbon representation. FFAR1-apo is in gray, FFAR1 in the FFAR1-γ-linolenic-acid complex is in cyan, and the γ-linolenic acid is shown as purple stick. Residues forming strong interactions with the γ-linolenic acid are shown as cyan stick, and hydrogen bonds are shown as yellow dashed lines. (C) Comparison of the intracellular helix movements in the FFAR1-apo (in gray) and FFAR1-γ-linolenic-acid (in cyan) structures.

To further understand the mechanism behind the difference in the downstream signaling of FFAR1 induced by the two allosteric epimers, we first analyzed the dynamic differences of FFAR1 between the FFAR1-apo and endogenous ligand γ-linolenic acid bound system. Compared with the conserved TM region, the loop region of FFAR1 in both systems experienced a much larger fluctuation during the simulations, especially for the ICL2 region (Figure 6A). In FFAR1-apo, ICL2 is disordered and shows large fluctuations. However, in the FFAR1-γ-linolenic-acid complex, ICL2 forms a short helix, which is consistent with what is observed in the X-ray crystal structure (5KW2).^12^ The difference in the ICL2 structure is likely caused by the fact that in the FFAR1-γ-linolenic-acid complex, the formation of a hydrogen bond between the carboxylate moiety of the γ-linolenic acid and Y114^ICL2^ of the FFAR1 stabilizes the helix of ICL2. In addition, the γ-linolenic acid can form hydrogen bonds with Y44^2.42^ and S123^4.42^ and extensive hydrophobic interactions with residues A98^3.44^, A99^3.45^, A102^3.48^, L106^3.52^, V134^4.53^, and P194^5.50^ at site 2 (Figure 6B), which to some extent stabilize the TM3 and TM4 around the binding site (Figure 6A). Rearrangement of the helices in a GPCR is important for its activation.^39^ From Figure 6C, we can see that the binding of the γ-linolenic acid triggers extensive rearrangement of the TM helices, especially for those bound directly to the G-protein, such as TM3, TM6, and TM7. The deflection of the TM helices to the outside of the helical bundle promotes the opening of the G-protein binding site, which is favorable for G-protein binding.

**Figure 7:**
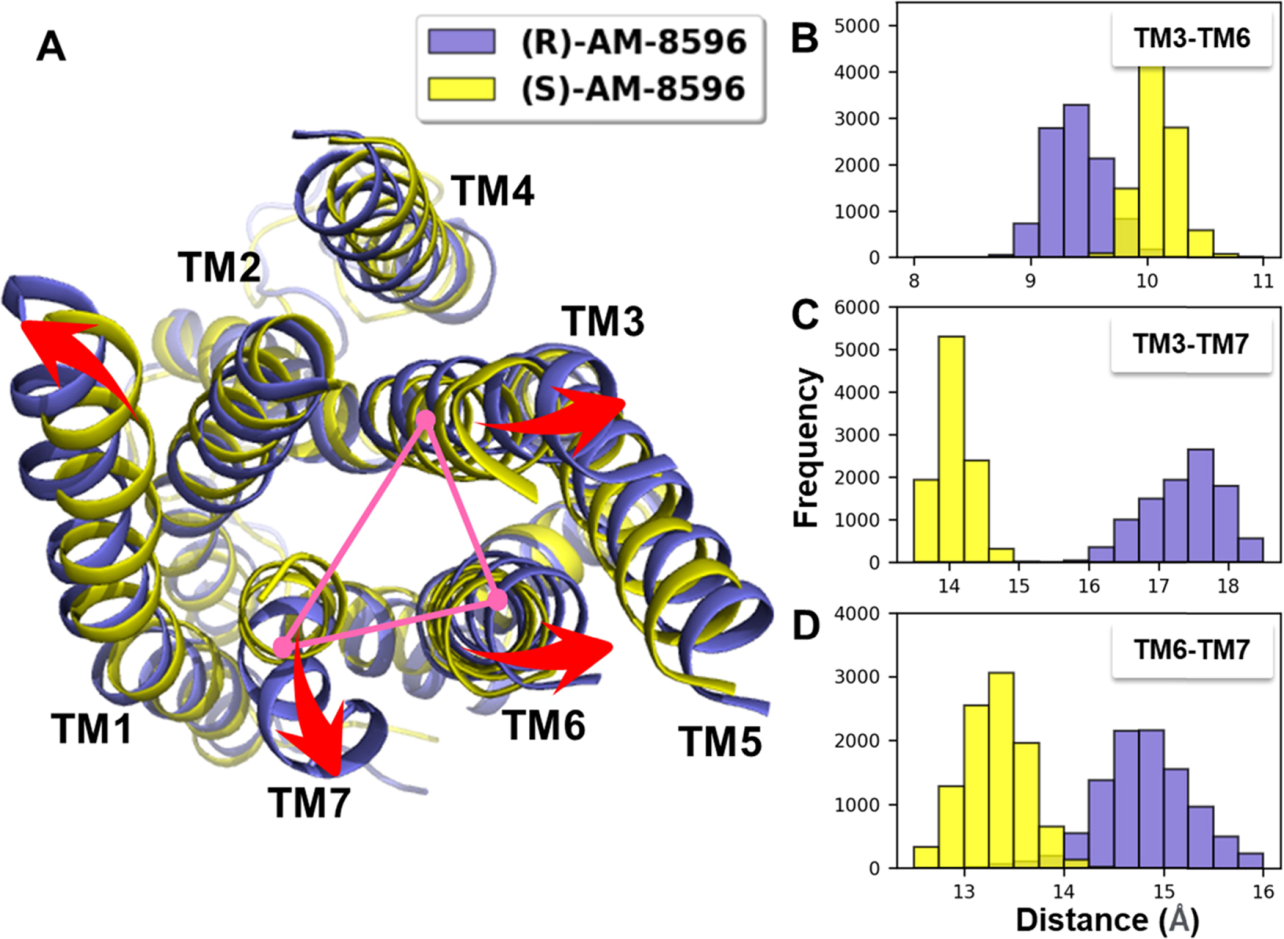
Helix movements of the agonist-bound FFAR1. The FFAR1 in the (*R*)-AM-8596- and (*S*)-AM-8596-bound systems are shown in purple and yellow, respectively. (A) Conformational changes of the helices in the two agonist-bound FFAR1 systems. (B–D) Distributions of the TM helix distances TM3–TM6, TM3–TM7, and TM6–TM7 in FFAR1.

As mentioned above, the binding of the endogenous ligand γ-linolenic-acid induces significant conformational changes in FFAR1. Here, we further investigate if the allosteric agonists (*R*,*S*)-AM-8596 could induce similar conformational changes in FFAR1. Compared to the FFAR1-apo system, the binding of the allosteric agonists (*R*,*S*)-AM-8596 could induce a helix rearrangement of FFAR1 (Figure S4A). However, there are some differences in the conformational changes of FFAR1 induced by the full agonist (*R*)-AM-8596 and the partial agonist (*S*)-AM-8596. It can be found that the *B*-factors of the intracellular helical bundle for the full agonist (*R*)-AM-8596-bound system are much higher than those for the partial agonist (*S*)-AM-8596-bound system (Figure S5), and the intracellular helices in the two agonist-bound systems undergo considerable movements. In the (*S*)-AM-8596-bound system, several key helices TM3, TM5, TM6, and TM7 were deflected outward by 0.7, 0.9, 2.5, and 1.6 Å, respectively, while in the (*R*)-AM-8596-bound system, the deflection distances of these helices correspond to1.8, 3.3, 3.4, and 3.0 Å. It can be found that the movements induced by the full agonist (*R*)-AM-8596 are more significant than those by the partial agonist (*S*)-AM-8596. Because TM3, TM6, and TM7 are in direct contact with the G-protein and play a critical role in the activation of a GPCR,^40^ we plotted the distributions of the distances between the three helices to characterize the extent of openness of the G-protein binding site (Figure 7). The result shows that in the full agonist (*R*)-AM-8596-bound system, the average distances are 9.39 Å for TM3–TM6, 17.39 Å for TM3–TM7, and 14.83 Å for TM6–TM7. In the partial agonist (*S*)-AM-8596-bound system, the average distances are 10.09 Å for TM3–TM6, 14.07 Å for TM3–TM7, and 13.31 Å for TM6–TM7. By analyzing the SASA of the G-protein binding site, we found that the G-protein binding site of FFAR1 in the full agonist (*R*)-AM-8596-bound system is larger than that in the partial agonist (*S*)-AM-8596-bound system (Figure S3). Compared with (*S*)-AM-8596, the binding of (*R*)-AM-8596 can promote the opening of the G-protein binding site to a greater extent.

**Figure 8:**
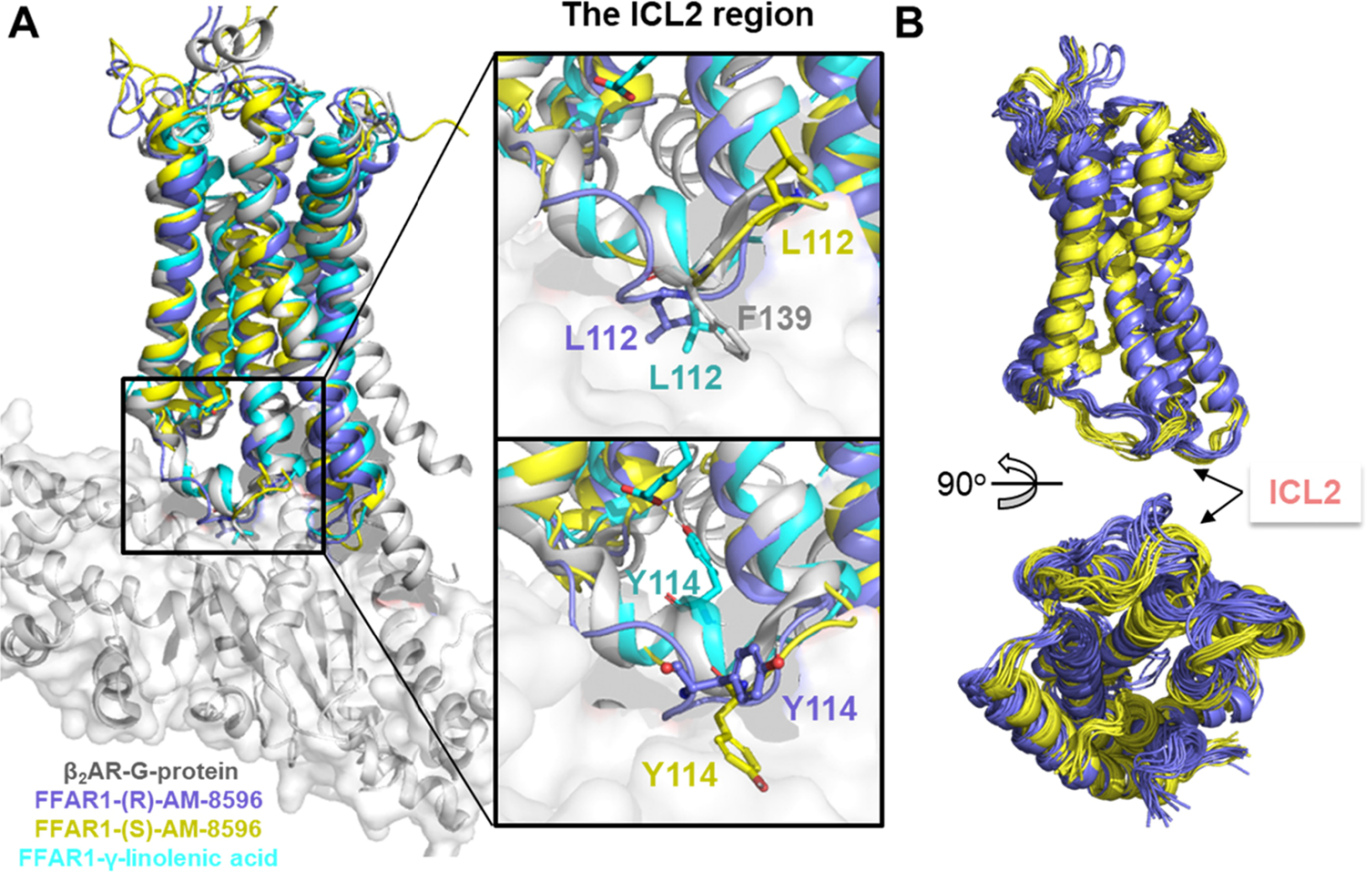
Conformation of ICL2 in FFAR1. (A) Superposition of the FFAR1 structures in different systems, together with the active-state β_2_AR in complex with the Gs-protein. The ICL2 region is highlighted in the middle subplot. (B) Ensemble of 20 representative conformations extracted from the MD simulations of the full agonist (*R*)-AM-8596-bound (in purple) and partial agonist (*S*)-AM-8596-bound (in yellow) systems, with the conformations extracted every 50 ns during the last 500 ns of the simulation.

Interestingly, in addition to the differences in the movements of the TM helices of FFAR1, significant conformational differences were also observed on ICL2 in different systems. ICL2 is a key interface element that contacts helix 5 on the G-protein and the TM3 and TM5 in the activated state of a GPCR.^41^ In the crystal structure of FFAR1-compound 1 complex (PDB ID: 5KW2), the stabilization of ICL2 by the full agonist compound 1 bound at site 2 could explain the enhancement of the pathway of the G-protein coupling to Gs-cAMP.^12^ From our simulations, we found that the endogenous ligand γ-linolenic acid bound at site 2 can also stabilize the short helical conformation of ICL2 through forming hydrogen-bond interactions with Y114^ICL2^ (Figure 6B). However, in the (*R*)-AM8596-bound, (*S*)-AM-8596-bound, and FFAR1-apo systems, in which no ligand is bound at site 2, the hydrogen-bond interactions that stabilize ICL2 no longer exist (Figure 8A), which is consistent with what have been observed in structure 4PHU.^11^ By analyzing the secondary structure of ICL2, we found that among the four systems, the residues involved in the helix formation accounted for the highest percentage (68%) in the FFAR1-γ-linolenic-acid system and the lowest percentage (20%) in the FFAR1-apo system. The percentage of the residues involved in the formation of the helix of ICL2 in the FFAR1-(*R*)-AM-8596 system (34%) is higher than that in the FFAR1-(*S*)-AM-8596 system (28%). Although the binding of the agonists (*R*)-AM-8596 and (*S*)-AM-8596 at site 1 of FFAR1 cannot stabilize the short helix conformation of ICL2, it promotes the formation of the α-helix of ICL2 to a certain extent, which indicates that the binding of the agonists (*R*,*S*)-AM-8596 could affect the conformation of ICL2 (Figure S4B). In the (*R*)-AM-8596-bound system, the deflection of ICL2 to the outside of the helical bundle (Figure 8B) further increases the G-protein binding site, which is favorable for the binding of the G-protein.

In the activated state of the β2AR-G-protein complex (Figure 8A), the residue F139^ICL2^ of β2AR forms important hydrophobic interactions with the Gs-protein. A previous work on mutagenesis suggests that a hydrophobic amino acid on ICL2 plays a key role in efficient GPCR-G-protein coupling.^42^ The position of the hydrophobic residue L112^ICL2^ in FFAR1 is equivalent to F139^ICL2^ of β2AR. The cAMP accumulation assays in FFAR1 with the L112A^ICL2^ mutant indicated that no observable Gs-protein stimulation was detected with the full agonist compound 1 concentration up to micromolar. Because L112^ICL2^ is a key hydrophobic residue at the interface between FFAR1 and the G-protein, we analyzed the conformations of L112^ICL2^ in different systems. We found that the conformation of L112^ICL2^ in the full agonist (*R*)-AM-8596-bound system is very similar to that in the endogenous ligand γ-linolenic acid bound system. Moreover, the position of L112^ICL2^ in FFAR1 is rather similar to that F139^ICL2^ in the active β2AR (Figure 8A), which is favorable for G-protein binding. Similar to the bulky hydrophobic side chain of F139^ICL2^, the side chain of L112^ICL2^ in the (*R*)-AM-8596-bound system faces the hydrophobic surface of the Gα-protein, which is favorable for the G-protein binding. However, in the (*S*)-AM-8596-bound system, L112^ICL2^ is completely away from the region for the G-protein binding and the polar side chain of Y114^ICL2^ faces the region for the G-protein binding (Figure 8A), which is unfavorable for the coupling of the G-protein. These structural differences may explain the different activation effects produced by the full agonist (*R*)-AM-8596 and the partial agonist (*S*)-AM-8596 and can most likely affect the binding of the G-protein and consequently the downstream signal transduction.

### 3.4 Analysis of Allosteric Communication

**Figure 9:**
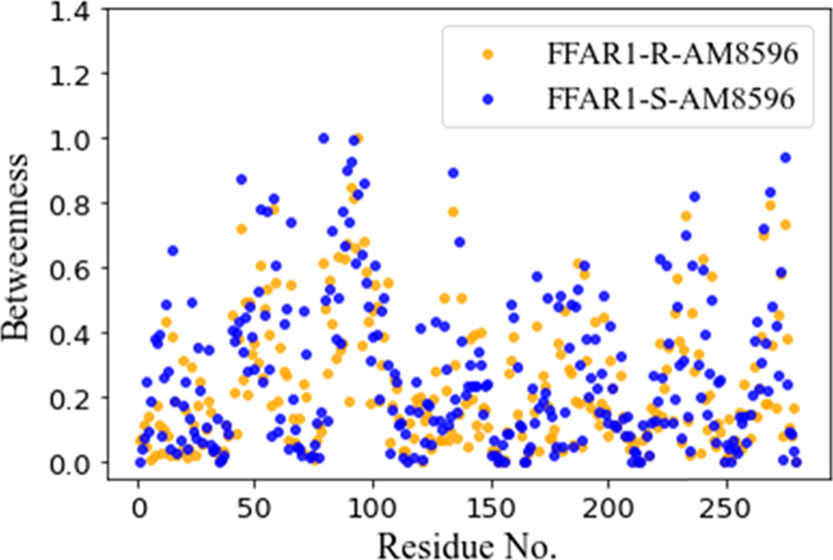
Residue-based betweenness profiles of the FFAR1 structures in the full agonist (*R*)-AM-8596-bound conformation (in orange) and partial agonist (*S*)-AM-8596-bound conformation (in blue).

As an important signaling protein, a GPCR contains three functional regions: the triggering region for the ligand binding, the allosteric linking core, and the G-protein coupling region.^43^ The binding of a ligand to the receptor can regulate the molecular switch within it, and through the linking core, the resulting conformational change is transmitted to the G-protein-coupled region, thereby leading to the activation of the G-protein.^44^ Betweenness of a residue, an important metric to characterize the centrality of residues, is defined as the ratio of the number of shortest paths through the residue to the total shortest paths in the network and represents a global centrality measure of the residue. Residues with high centrality play a key role in allosteric signal transduction.^45^ Studies on kinases have shown that the stability of a protein structure is related to the number of high centrality residues.^45^ We have calculated the residue betweenness of FFAR1 in different systems. We found that the residues with high betweenness are highly consistent with the residues that mediate class A GPCR signaling identified in previous studies.^43,46^ The residue betweenness in the FFAR1-(*S*)-AM-8596 complex is higher than that in the FFAR1-(*R*)-AM-8596 complex (Figure 9), indicating that the structure of FFAR1 in the (*S*)-AM-8596-bound system is more stable.

**Figure 10:**
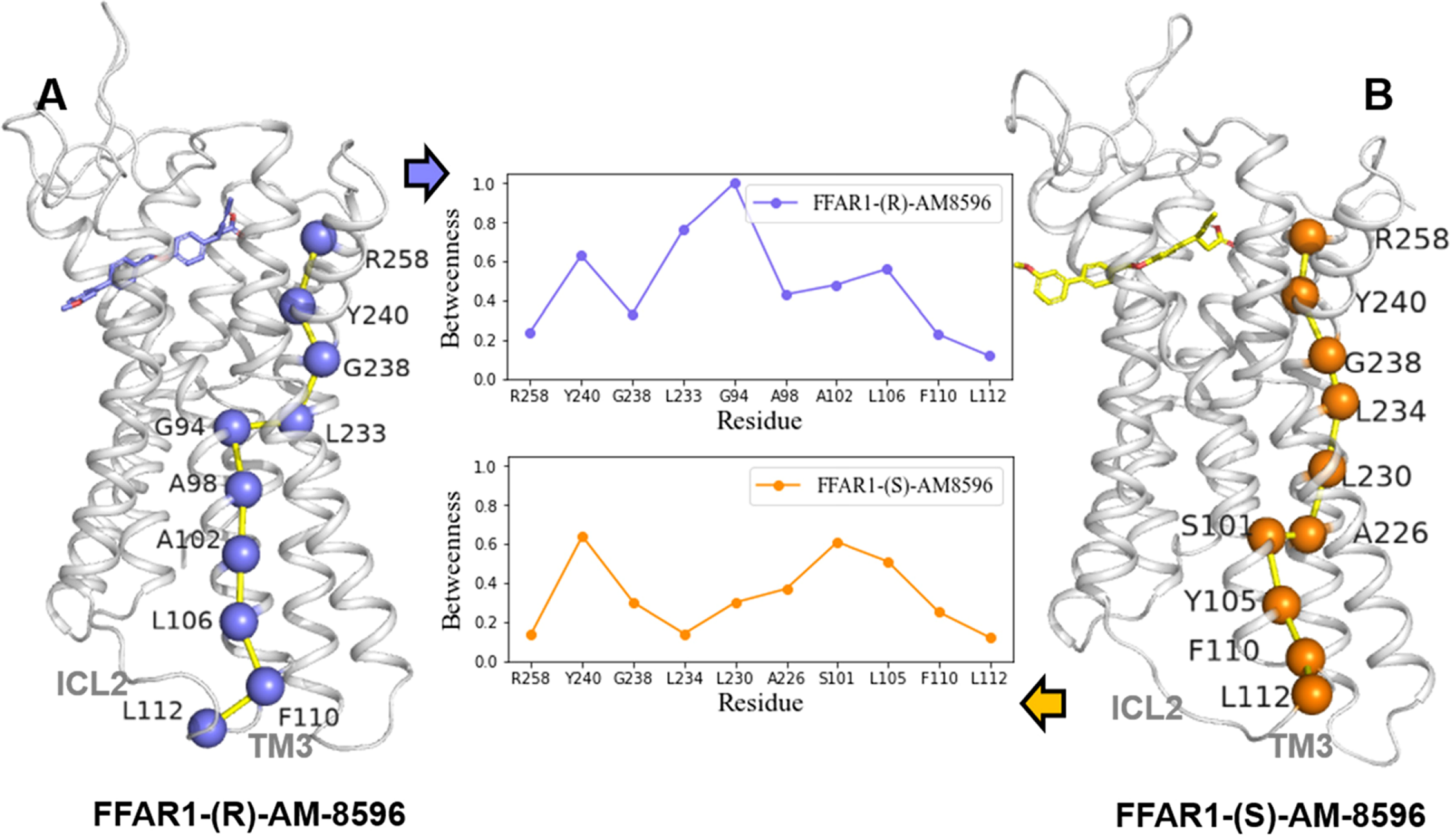
Shortest path analysis of the FFAR1-(*R*)-AM-8596 (A) and FFAR1-(*S*)-AM-8596 (B) systems. The betweenness of the residues involved in the shortest path is shown in the middle subgraph.

To explore if an agonist binding allosterically regulates the conformational change of ICL2, we applied the network-based analysis method and identified the shortest path from each of the key residues in the binding pocket of FFAR1 to ICL2 for the systems with FFAR1 bound to the agonists (*R*,*S*)-AM-8596. For a pair of selected residues, NAPS can figure out all the possible shortest paths. Because R258^7.35^ is a key residue in the binding pocket of FFAR1 and L112^ICL2^ is a critical residue on ICL2 that affects the G-protein coupling, we selected residues R258^7.35^ and L112^ICL2^ for the shortest path analysis. Previous studies have shown that high centrality residues involved in the regulation of allosteric signaling can be supported by the adjacent nodes that provide sufficient robustness and functional redundancy for failures caused by targeted or random mutations. Residues with high centrality are more prone to connecting with each other, forming resilient and rapid communication paths.^45^ Here, we only considered the shortest paths consisting of residues with high betweenness. We found that the residue betweenness of the shortest path between R258^7.35^ and L112^ICL2^ in the (*R*)-AM-8596-bound system was on average higher than in the (*S*)-AM-8596-bound system (Figure 10). Therefore, compared with the partial agonist (*S*)-AM-8596, the full agonist (*R*)-AM-8596 has a higher probability of regulating the conformation of L112^ICL2^ through the pathway formed between R258^7.35^ and L112^ICL2^. A previous work on mutagenesis and functional experiments in FFAR1 suggested that residues R258^7.35^, Y240^6.51^, and L112^ICL2^ play critical roles in the allosteric regulation and activation of FFAR1 by agonists.^6,11,12^ Also, the TM3 and ICL2 regions traversed by the shortest path play an important role in the G-protein coupling. These results indicate that the full agonist (*R*)-AM-8596 may regulate the side chain conformation of L112^ICL2^ through a more robust path and promote the binding of the G-protein, thereby facilitating the (*R*)-AM-8596 activation. Our results reveal that different agonists can not only modulate differently the opening of the G-protein binding site by affecting the rearrangement of helices TM3, TM6, and TM7 but also affect differently the conformation of ICL2. These observations imply that the conformational change of ICL2 is crucial to the signal transduction of FFAR1.

## 4 Conclusions

In this work, by using molecular modeling, free energy calculation, and residue network analysis methods, we reveal the molecular mechanism of the selectivity of FFAR1 modulators, which are a pair of optical isomers, with one acting as a partial agonist and the other as a full agonist. The structural details uncovered in this work provide a new insight into the key step in the stereoselectivity of agonist-induced FFAR1 activation. We found that the full agonist (*R*)-AM-8596 has weaker electrostatic interactions with the receptor than the partial agonist (*S*)-AM-8596, which indicates that (*R*)-AM-8596 is less stable in the receptor. In the full agonist-bound system (FFAR1-(*R*)-AM-8596), the propynyl moiety in the chiral center of (*R*)-AM-8596 faces ECL2 and forms hydrophobic interactions with L158^ECL2^ and L171^ECL2^, which makes (*R*)-AM-8596 less stable at the binding site. The instability of the full agonist (*R*)-AM-8596 disrupts the interactions between the residues in the receptor binding pocket to a greater extent, which further drives the movements of the TM3, TM6, and TM7 and promotes the opening of the G-protein binding site. Furthermore, the binding of the full agonist (*R*)-AM-8596 not only induces the outward deflection of ICL2 to further promote the opening of the G-protein binding site of FFAR1 but also regulates the conformation of the key residue L112^ICL2^ toward the region for the G-protein binding and facilitates the G-protein binding. However, in the partial agonist (*S*)-AM-8596-bound system, (*S*)-AM-8596 forms strong salt bridge interactions with R183^5.39^ and R258^7.35^, and the propynyl group of (*S*)-AM-8596 is deflected out of the helical bundle and trapped in between TM4 and TM5; thus, the conformation of (*S*)-AM-8596 becomes more stable. (*S*)-AM-8596 also forms strong hydrogen-bond interactions with Y240^6.51^, which further increases the density of the residue interaction network within the receptor and reduces the flexibility of the receptor structure. Thus, we believe that the full agonist (*R*)-AM-8596 promotes the disruption of the residue interaction network around the binding pocket of the receptor, and the resulting large-scale movements of TM helices, together with the conformational change of ICL2, enlarge the G-protein binding site of FFAR1, which in turn triggers the full activation of FFAR1. Our results suggest that the agonism of this pair of diastereomers is mainly dependent on the difference in the agonist-induced receptor structural changes. These molecular details may also explain why different agonists trigger different downstream signal transduction.

In addition to the above-mentioned structural differences, the two agonists share a similar binding mode. Anchoring of an agonist by R183^5.39^ and R258^7.35^ via strong electrostatic interactions is a key step in the agonist activation of FFAR1, and the charged group on the ligand head is thus necessary for maintaining the activity. We believe that the strong π-π stacking interaction formed by an agonist with F87^3.33^ and F142^4.61^ increases the binding affinity of the agonist to the receptor. As such, our findings are helpful for designing FFAR1-specific agonists.

In this work, the residue interaction network method was used to study the difference in the effect of receptor modulators on the receptor conformation. Through the obtained residue interaction network, we can find key differences that influence the ligand selectivity. The partial agonist (*S*)-AM-8596 with higher binding affinity to the receptor FFAR1 stabilizes the residue network within the receptor, which results in a rigid receptor structure and hinders the receptor conformational transitions. However, the full agonist (*R*)-AM-8596 has a low binding affinity to the receptor, which disrupts the residue interaction network of the receptor, results in a more flexible protein structure, and promotes the receptor to adopt a more active conformation. In particular, we believe that the mechanism of different agonists on the stability of the FFAR1 residue interaction network is likely also valid for other GPCR systems. The structural changes uncovered in this work provide valuable insights into the ligand stereoselectivity and biased signaling for other GPCRs. We believe the strategy adopted in this study can also be used for studying the ligand selectivity of other systems.

## Supporting Information Available

Overview of thermodynamic cycle; experimental setup in the free energy calculation; comparison of SASA distributions, dynamic differences and *B*-factors of FFAR1 in different systems; and the decomposition terms of binding free energy (PDF)

## Notes

The PDB files used in this study were downloaded from the RCSB protein data bank (https://www.rcsb.org/). Schrödinger Suite Glide at https://www.schrodinger.com/products/glide is free for licensed users and evaluation purposes. Schrödinger Suite Maestro is free for academic use and evaluation purposes at https://www.schrodinger.com/products/maestro. The protein–ligand interaction analysis tool PLIP is available at https://plip-tool.biotec.tu-dresden.de/plip-web/plip/index. The open-source free energy analysis tool alchemical-analysis.py is available on GitHub as part of the pymbar package (http://github.com/choderalab/pymbar). The open-source molecular visualization program VMD can be accessed from https://www.ks.uiuc.edu/Development/Download/download.cgi?PackageName=VMD. The open-source software platform Cytoscape can be accessed from https://cytoscape.org/. The protein network analysis tool RING is available at http://old.protein.bio.unipd.it/ring/, and NAPS is available at https://bioinf.iiit.ac.in/NAPS/index.php. The parameter settings in the simulations and the detailed descriptions of the software along with the workflow can be found in the Materials and Methods section and Supporting Information.

## Abbreviations

ECL: extracellular loop
FFAR1: free fatty acid receptor 1
GIP: glucose-dependent insulinotropic peptide
GLP-1: glucagon-like peptide 1
GPCRs: G-protein-coupled receptors
ICL: intracellular loop
MD: molecular dynamics
PLIP: protein ligand interaction profiler
ProPrep: protein preparation wizard
SASA: solvent accessible surface area
T2D: type 2 diabetes
TM: transmembrane.
