# Supplementary material for: Mechanistic Studies on the Stereoselectivity of FFAR1 Modulators
Source: J Chem Inf Model. 2022 Jul 25;62(15):3664–75. doi: 10.1021/acs.jcim.2c00417 (PMC9364411; doi:10.1021/acs.jcim.2c00417)
Supplement: Supplementary file 1 — ci2c00417_si_001.pdf [file ci2c00417_si_001.pdf]

Supporting Information

Mechanistic studies on the stereoselectivity of FFAR1 modulators

Dan Teng[a,b], *Yang Zhou*[b,c], *Yun Tang*[a], *Guixia Liu*[a]*, *Yaoquan Tu*[b]*

[a]Shanghai Frontiers Science Center of Optogenetic Techniques for Cell Metabolism, Shanghai Key Laboratory of New Drug Design, School of Pharmacy, East China University of Science and Technology, Shanghai 200237, China

[b]Department of Theoretical Chemistry and Biology, School of Engineering Sciences in Chemistry, Biotechnology and Health (CBH), KTH Royal Institute of Technology, SE-106 91 Stockholm, Sweden

[c]School of Pharmacy, Jinan University, 601 Huangpu Avenue West, Guangzhou 510632, China

* Corresponding authors. Tel: +46 8 790 96 45; +86-21-64250811.

E-mail addresses: yaoquan@kth.se (Y. Tu), gxliu@ecust.edu.cn (G. Liu).

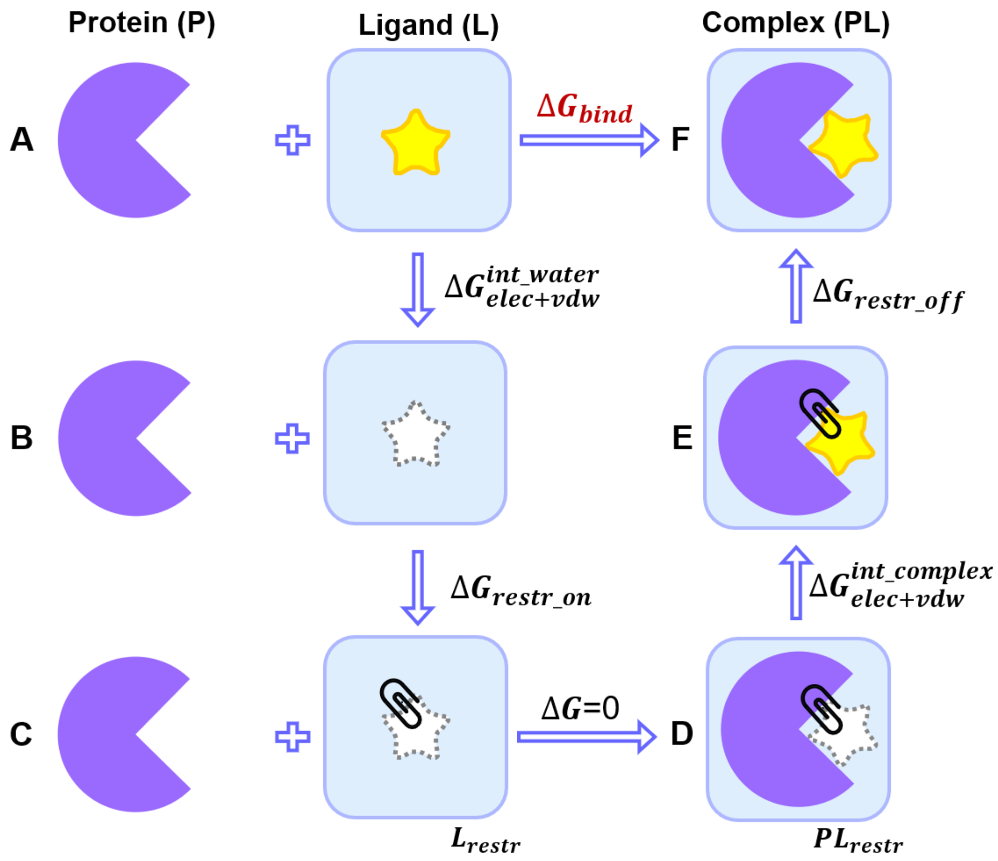

Figure S1. Thermodynamic cycle for binding free energy calculations. In this cycle, the circular shape with a breach represents the protein, the breach presents the binding site, and the star represents the ligand. The fully interacting ligand in water in the upper left (A, the yellow star) is transformed into a non-interacting ligand (B, the white star with dotted border) through a series of intermediate states for calculating $\Delta G^{int\_water}_{elec+vdw}$. The ligand is then constrained while still not interacting with the environment (C, the white dotted star with paper clip), giving ΔG_{restr_on}. The above state is equivalent to a non-interacting ligand restrained within the binding pocket (D). In the complex, the electrostatic and vdW interactions between the ligand (restrained and non-interacting) and the protein have turned back on again (E), providing $\Delta G^{int\_complex}_{elec+vdw}$. Then, the restraints between the protein and the ligand are removed (F), and the cycle ends.

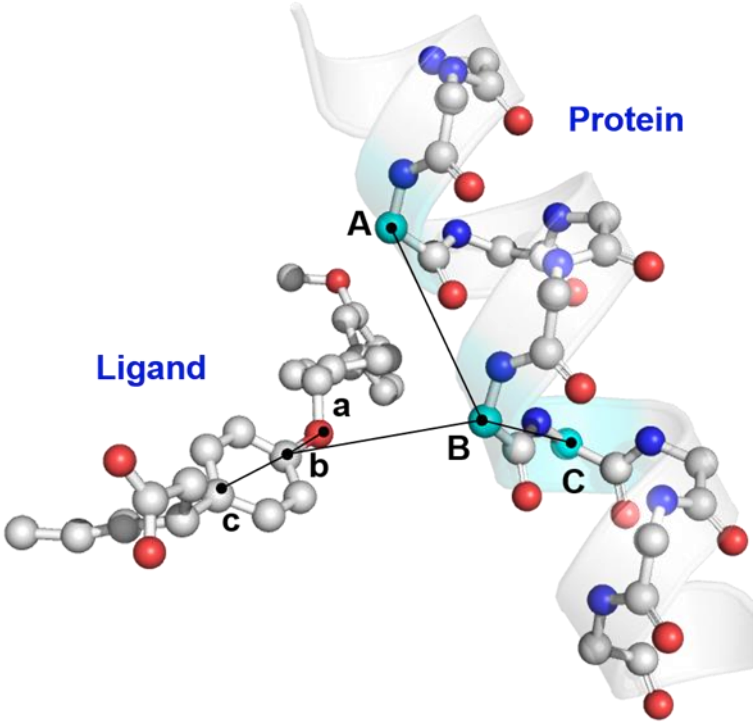

Figure S2. Ligand and protein atoms selected for the restraints.

*The distance, angles and dihedral angles are respectively defined as:

d: A-a

θ_A: a-b-B

θ_B: C-B-b

φ_A: c-b-B-C

φ_B: a-b-B-A

φ_C: A-B-C-b

Table S1. Selected atoms, and the reference distances and angles for the restraints used to keep the decoupled ligand at the binding site of the protein.

Ligands	Protein Reference atoms	d (nm)	θ_A (degree)	θ_B (degree)	φ_A (degree)	φ_B (nm)	φ_C (degree)
(R)AM-8596	A: A83(Cα)<br>B: F87(Cα)<br>C: F88(Cα)	0.62	80	120	151.7	61.1	-70
(S)AM-8596	A: A83(Cα)<br>B: F87(Cα)<br>C: F88(Cα)	0.65	100.6	95.9	146.3	73.8	-51

Table S2. Electrostatic and van der Waals interactions between a ligand and FFAR1.

Ligand	$-\Delta G_{elec+vdw}^{int\_complex}$	Electrostatic	van der Waals
(R)-AM-8596	-121.602 ± 0.524	-104.799 ± 0.469	-16.803 ± 0.234
(S)-AM-8596	-123.153 ± 0.310	-106.097 ± 0.152	-17.056 ± 0.270

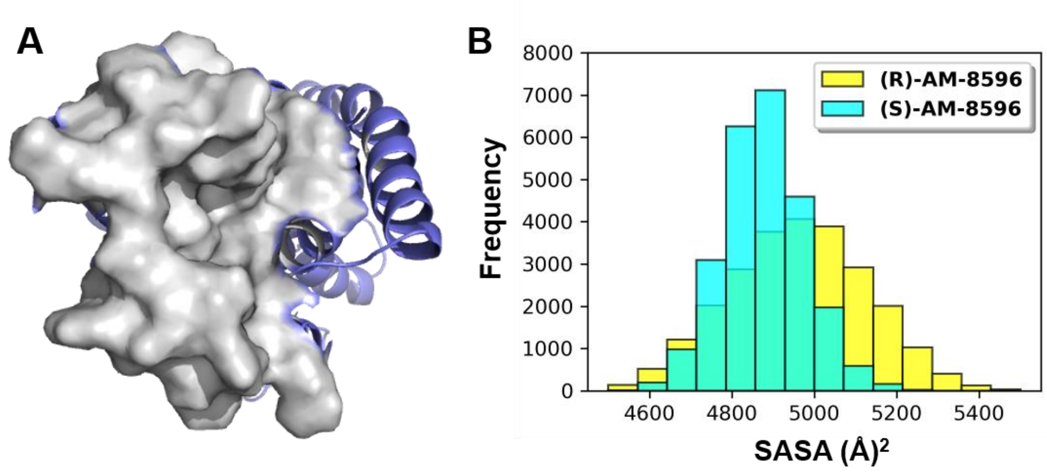

Figure S3. SASA distributions of the G-protein binding site of FFAR1. (A) Equivalent residues in FFAR1 determined according to the Gα-protein binding site of the β2 adrenergic receptor. (B) SASA distributions of the Gα-protein cavity in the full agonists bound system (FFAR1-(R)-AM-8596) and the partial agonist bound system (FFAR1-(R)-AM-8596). 45 equivalent residues were used to study the SASA distributions, which are P40, S41, L42, Y44, A45, A99, L100, A102, G103, R104, L106, G107, A108, P111, L112, Y114, Q115, R118, R119, Y122, S123, Y202, C205, L206, A208, L209, S212, G213, L214, T215, R217, R218, K219, R221, A222, W224, V225, A226, G228, A229, V275, T276, G277, Y278.

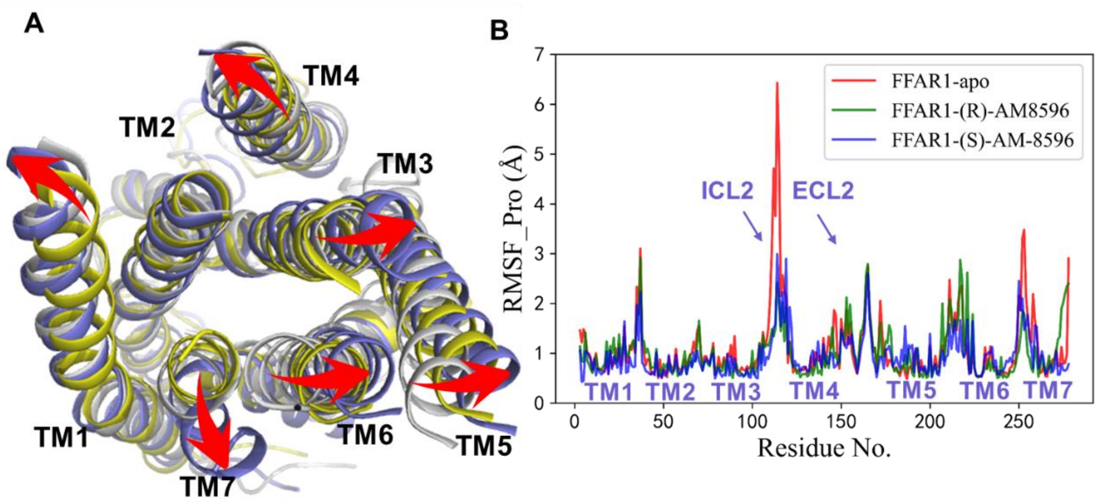

Figure S4. Dynamic differences between the resting receptor and (R,S)-AM-8596-bound receptor. (A) Superposition of the FFAR1 structures in the FFAR1-apo (in gray), FFAR1-(R)-AM-8596 (in purple), and FFAR1-(R)-AM-8596 (in yellow) systems. (B) RMSF values of the FFAR1 Cα-atoms obtained from the MD simulations.

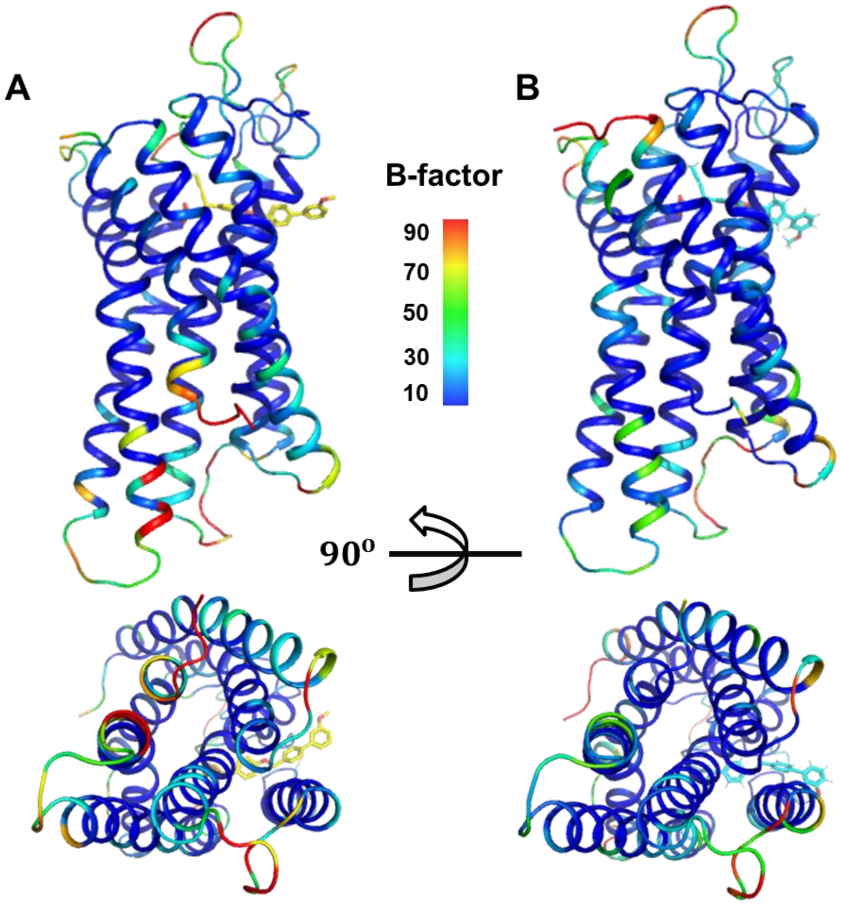

Figure S5. B-factors (Å^2) of FFAR1 in FFAR1-(R)-AM-8596 (A) and FFAR1-(S)-AM-8596 (B).
